# Time-to-surgery paradigms: wait time and surgical outcomes in critically Ill patients who underwent emergency surgery for gastrointestinal perforation

**DOI:** 10.1186/s12893-024-02452-w

**Published:** 2024-05-17

**Authors:** Junghyun Lee, Chami Im

**Affiliations:** 1 Department of Surgery, Yongin Severance Hostpital, Yongin, Korea; 2 Department of Surgery, Seoul National University Bundang Hospital, Seongnam, Korea; 3 Seoul National University College of Medicine, Seoul, Korea; 4 Yonsei University College of Medicine, Seoul, Korea

**Keywords:** Acute Abdomen, Intestinal Perforation, Acute care surgery, Perioperative care

## Abstract

**Background:**

Waiting time for emergency abdominal surgery have been known to be linked to mortality. However, there is no clear consensus on the appropriated timing of surgery for gastrointestinal perforation. We investigated association between wait time and surgical outcomes in emergency abdominal surgery.

**Methods:**

This single-center retrospective cohort study evaluated adult patients who underwent emergency surgery for gastrointestinal perforations between January 2003 and September 2021. Risk-adjusted restricted cubic splines modeled the probability of each mortality according to wait time. The inflection point when mortality began to increase was used to define early and late surgery. Outcomes among propensity-score matched early and late surgical patients were compared using percent absolute risk differences (RDs, with 95% CIs).

**Results:**

Mortality rates began to rise after 16 h of waiting. However, early and late surgery groups showed no significant differences in 30-day mortality (11.4% vs. 5.7%), ICU stay duration (4.3 ± 7.5 vs. 4.3 ± 5.2 days), or total hospital stay (17.4 ± 17.0 vs. 24.7 ± 23.4 days). Notably, patients waiting over 16 h had a significantly higher ICU readmission rate (8.6% vs. 31.4%). The APACHE II score was a significant predictor of 30-day mortality.

**Conclusions:**

Although we were unable to reveal significant differences in mortality in the subgroup analysis, we were able to find an inflection point of 16 h through the RCS curve technique.

**Trial registration:**

Formal consent was waived due to the retrospective nature of the study, and ethical approval was obtained from the institutional research committee of our institution (B-2110–714-107) on 6 October 2021.

## Introduction

Surgical emergency is a medical emergency for which immediate surgical intervention is the only way to solve the problem successfully [1]. Gastrointestinal (GI) perforation is one of the most common intra-abdominal surgical emergencies [2, 3]. GI perforation is a defect in the wall of the gastrointestinal tract due to various mechanisms [2, 4]. Various factors have been found to cause GI perforations, including ulcerative lesions, ischemia, obstruction, infection, cancer, trauma, and endoscopic intervention [2]. Regardless of the cause, spilled intestinal contents could cause intra-abdominal infections that often lead to sepsis and septic shock. This represents a major life-threatening condition with high morbidity and mortality rates, requiring surgical intervention [5]. The overall mortality rate of emergency surgery for GI perforation has been reported to be 16.9–30%. Despite advances in surgical and medical treatments, this rate remains relatively higher than that in patients undergoing elective surgery for GI cancer [6–9]. Several studies have identified the prognostic factors associated with morbidity and mortality in patients with GI perforation. However, these are limited to anatomic subspecialties. In addition, few studies have been conducted including patients with GI perforations who required acute care surgery.

Emergency operations from all surgical disciplines should be evaluated by a surgeon and scheduled within an agreed time frame established on evidence-based data of outcomes related to the time elapsed from diagnosis to surgery [10, 11]. According to the international guidelines by the surviving sepsis campaign, smaller studies have suggested that source control within 6–12 h was advantageous [10, 12]. Other studies of patients with gastric and duodenal ulcer perforation recommended its resolution within 12–24 h [6, 7]. Although the literature exists regarding the optimal timing of various surgical interventions, data are still limited and require high-quality follow-up research to conclusively issue a recommendation for the optimal timing of surgical source control.

Therefore, the primary aim of this study was to statistically assess the relationship between the time from admission to surgery (TTS) and 30-day outcomes and determine the optimal time from admission to initiate surgical intervention for favorable outcomes. The secondary aim was to identify the predictors associated with 30-day mortality in critically ill patients who underwent emergency surgery for GI perforation.

## Materials and methods

**Fig. 1:**
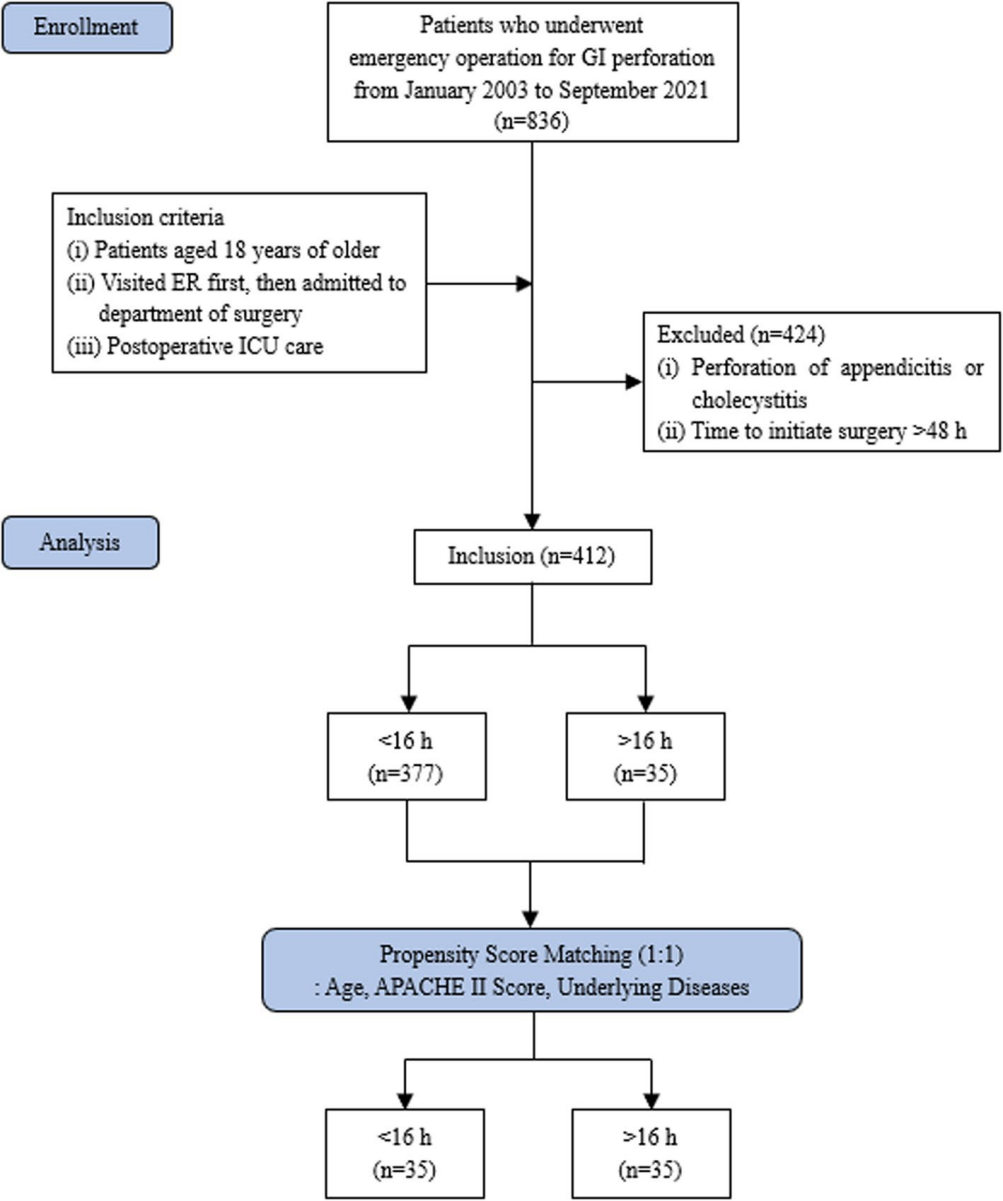
Study flowchart of patient selection and 1:1 matching criteria

This retrospective cohort study was conducted at Seoul National University Bundang Hospital. Between January 2003 and September 2021, 836 patients underwent emergency surgery for GI perforation. The inclusion criteria included patients: aged 18 years or older, admitted to the department of surgery through the emergency room, and admitted to the intensive care unit (ICU) after surgery. Finally, 412 patients were included in this study. Patients with perforations due to appendicitis or cholecystitis were excluded from the study. Patients who underwent surgery > 48 h after admission were also excluded (Fig. 1).

Formal consent was waived due to the retrospective nature of the study, and ethical approval was obtained from the institutional research committee of our institution (B-2110–714-107) on 6 October 2021.

In this study, clinical data, laboratory test results, and operative findings were collected by reviewing the medical records, stored in the Bundang Hospital Electronic System for Total Care (BESTCare) at Seoul National University Bundang Hospital. A clinical data warehouse (CDW) application was used for data retrieval and analysis [13]. Demographics, factors associated with postoperative mortality within 30-day and those required to score Acute Physiology and Chronic Health Evaluation (APACHE) II score were evaluated. These factors comprised: age; sex; body mass index (BMI); Glasgow coma score (GCS); initial vital signs including mean arterial pressure (MAP), heart rate, respiratory rate, and body temperature; arterial pH; FiO2; PaO2; serum levels of sodium (Na), potassium (K), and creatinine (Cr); hematocrit (Hct); and white blood cell (WBC) count.

The primary endpoint of this study is to assess the relationship between the time from admission to surgery and 30-day mortality and determine whether there is a special inflection point that can determine the prognosis in surgical emergencies. The secondary aim is to identify the predictors associated with 30-day mortality.

A restricted cubic spline (RCS) curve model was used for the analysis of mortality rates according to the TTS. This method could express the odds ratio or hazard ratio as a cubic curve and be used for evaluating potential non-linear relationships [14]. We used this method to assess the association between TTS and probability of mortality.

Propensity score matching (PSM) was performed using the MatchIt package in R software. Covariates in the model for propensity scores included age, APACHE II score and comorbidities. A 1:1 matched analysis using nearest-neighbor matching with a caliper distance of 0.2 was performed based on the estimated propensity score of each patient. In all analyses, *P*-value of 0.05 or less was considered significant. All statistical analyses were performed using R Statistical Software version 4.2.2.

Fisher’s exact and chi-square tests were used for comparing between survivors and non-survivors. The Mann–Whitney U test and Student’s t-test were used to analyze continuous variables. Univariable logistic regression analysis was initially performed to assess individual associations between all covariates and postoperative mortality. Next, covariates with a *P*-value of < 0.1 from the univariable model were fitted into the backward stepwise regression model. Multivariable logistic regression was used for calculating odds ratio (OR) and 95% confidence interval (CI) and identifying independent factors associated with postoperative mortality. The results of the ROC analysis were presented as area under the curve (AUC) with 95% CI.

## Results

**Fig. 2:**
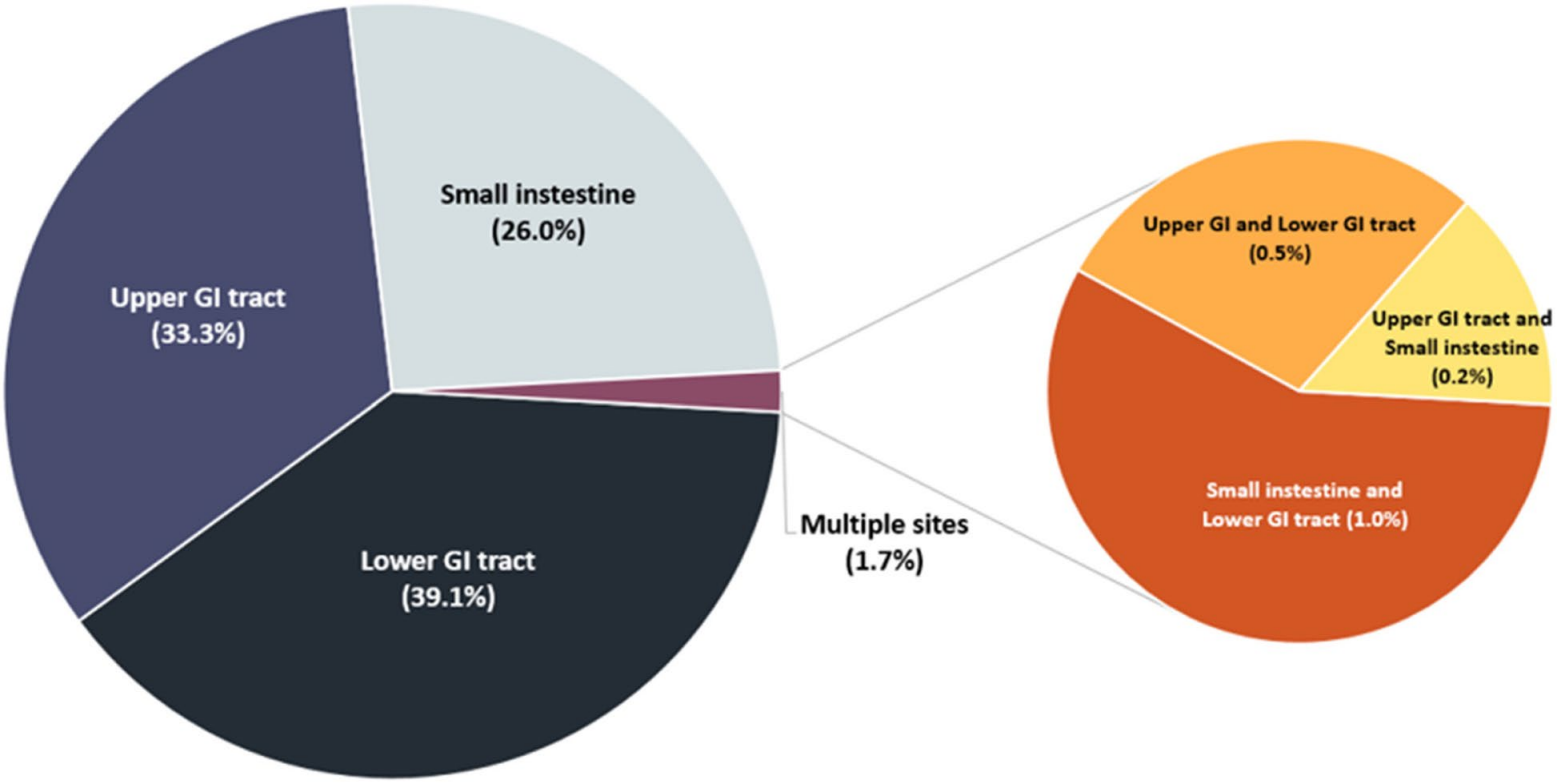
The anatomical location of GI perforation. *GI, gastrointestinal

Of the 412 patients, 56.17% were male. The mean age was 65.9 ± 16.2 years. The anatomical location of the GI perforation was: 39.1% in the lower gastrointestinal tract (LGI) including the colon, rectum, and anus; 33.3% in the upper gastrointestinal tract (UGI), from the distal esophagus to the duodenum; and 26% in the small intestine (Fig. 2). The incidence of multiple perforations was 1.7%, with simultaneous perforations in the small intestine and LGI tract occurring in 1.0%, followed by perforations in the UGI and LGI tracts in 0.5%, and perforations involving the UGI tract and small intestine in 0.2% of the cases. The mean TTS was 9.7 ± 7.8 h. The average operating time was 2.52 ± 1.21 h. The mean ICU stay was 4.4 ± 7.7 days and mean total hospital stay was 20.5 ± 25.0 days. In the non-survivors group, the mean time from admission to mortality was 9.5 ± 8.7 days. Three hundred seventy-six patients (91.01%) survived. However, 36 patients (0.09%) expired after surgery.

Based on 30-day mortality, the participants were allocated to two groups: survivors and non-survivors. The association between demographics and factors associated with postoperative 30-day mortality is presented in Table 1.

**Table 1:** Baseline demographics and univariable analysis of the factors associated with 30-day mortality

Variables	Survivors	Non-survivors	*P*-value
	(*n* = 376)	(*n* = 36)	
Sex (Male)	215 (57.2%)	16 (44.4%)	0.195
Age	65.0 ± 16.3	75.3 ± 11.3	< 0.001
Height (cm)	154.9 ± 23.6	156.2 ± 10.4	0.900
Body weight (kg)	58.8 ± 12.6	55.7 ± 11.3	0.199
BMI (kg/m^2^)	23.3 ± 9.6	30.9 ± 38.8	0.378
Operation time (h)	2.48 ± 1.18	2.91 ± 1.42	0.042
TTS (h)	9.7 ± 7.7	10.2 ± 9.3	0.701
ICU readmission	51 (13.6%)	9 (25.0%)	0.107
ICU stay (days)	4.2 ± 7.8	6.1 ± 6.5	0.161
Total hospital stay (days)	21.6 ± 25.8	9.6 ± 8.8	< 0.001
DM	70 (18.6%)	9 (25.0%)	0.479
Hypertension	167 (44.4%)	21 (58.3%)	0.154
Dialysis	11 (2.9%)	2 (5.6%)	0.716
Cardiac disease	48 (12.8%)	4 (11.1%)	0.982
Cerebrovascular disease	22 (5.9%)	3 (8.3%)	0.818
Cancer	111 (29.5%)	15 (41.7%)	0.186
Anatomical lesion of the perforation site			
UGI	132 (35.1%)	8 (22.2%)	0.169
Small bowel	102 (27.1%)	10 (27.8%)	1.000
LGI	142 (37.8%)	20 (55.6%)	0.056
APACHE II score	23.0 ± 8.7	40.3 ± 9.1	< 0.001
Lactate (mmol/L)	2.8 ± 3.2	6.7 ± 5.5	0.002
pH	7.4 ± 0.1	7.3 ± 0.2	0.005
HCO3 (mmol/L)	20.7 ± 4.2	16.3 ± 6.4	0.001
Hb (g/dL)	12.6 ± 2.6	11.7 ± 2.5	0.070
WBC (10^3^/µl)	11.7 ± 8.5	9.8 ± 8.1	0.200
CRP (mg/dL)	9.8 ± 11.4	13.7 ± 12.3	0.057
BUN (mg/dL)	25.6 ± 16.6	39.7 ± 31.8	0.012
Cr (mg/dL)	1.3 ± 1.1	1.8 ± 1.5	0.053
Procalcitonin (ng/mL)	17.9 ± 29.7	27.8 ± 38.8	0.448
Transfer from other hospitals	132 (35.1%)	11 (30.6%)	0.715

Data are presented as number (percentage) or mean (± standard deviation)

Abbreviations: *BMI* body mass index, *TTS* time to surgery, *ICU* intensive care unit, *DM* diabetes mellitus, *UGI* upper gastrointestinal, *GI* lower gastrointestinal, *APACHE II* Acute Physiology and Chronic Health Evaluation II, HCO3 bicarbonate, *Hb* hemoglobin, *WBC* white blood cells, *CRP* C-reactive protein, *BUN* blood urea nitrogen, *Cr* serum creatinine

The non-survivors had significantly older age (65.04 ± 16.31 versus 75.33 ± 11.32 years, *P* < 0.001), longer operating time (2.48 ± 1.18 versus 2.91 ± 1.42 h, *P* = 0.042) and shorter hospital stays (21.57 ± 25.79 versus 9.56 ± 8.79 days, *P* < 0.001) compared with the survivors. Sex (57.18 versus 44.44%, *P* = 0.195, in male), BMI (23.26 ± 9.61 versus 30.9 ± 38.84, *P* = 0.378), length of stay in the ICU (4.2 ± 7.82 versus 6.11 ± 6.54 days, *P* = 0.161), and TTS (9.67 ± 7.71 versus 10.2 ± 9.3 h, *P* = 0.701) were not statistically significantly different from 30-day mortality after surgery.

The non-survivors group showed higher APACHE II score (23.01 ± 8.66 versus 40.32 ± 9.16, *P* < 0.001); serum lactate (2.77 ± 3.18 versus 6.68 ± 5.52, *P* = 0.002); and BUN (25.58 ± 16.62 versus 39.7 ± 31.78, *P* = 0.012) compared with the survivors group. However, the non-survivors group showed a lower level of pH (7.39 ± 0.07 versus 7.3 ± 0.15, *P* = 0.005) and HCO3 (20.72 ± 4.25 versus 16.27 ± 6.39, *P* = 0.001) compared with the survivors group. Lower serum hemoglobin (Hb) levels (12.57 ± 2.62 versus 11.74 ± 2.5, *P* = 0.07); higher serum CRP (9.84 ± 11.35 versus 13.71 ± 12.26, *P* = 0.057); and Cr (1.3 ± 1.08 versus 1.82 ± 1.51, *P* = 0.053) levels were observed in the non-survivors group, without statistical significance. There was no significant difference between the two groups regarding the underlying diseases, such as hypertension, diabetes, cardiovascular disease, cerebrovascular diseases, and cancer. The anatomical location of perforation was also compared between the two study groups. There was no significant difference in perforation of the UGI tract (35.2% versus 22.22%, *P* = 0.166) or small intestine (28.86% versus 27.79%, *P* = 1.000). Although not statistically significant, in the LGI tract (37.77% vs. 55.56%, *P* = 0.056), there was a tendency for more LGI perforations in the non-survivors group. The number of patients transferred from other hospitals was 132 (35.1%) in the survivors group and 11(30.6%) in the non-survivors group, with no significant difference (*P* = 0.715).

**Fig. 3:**
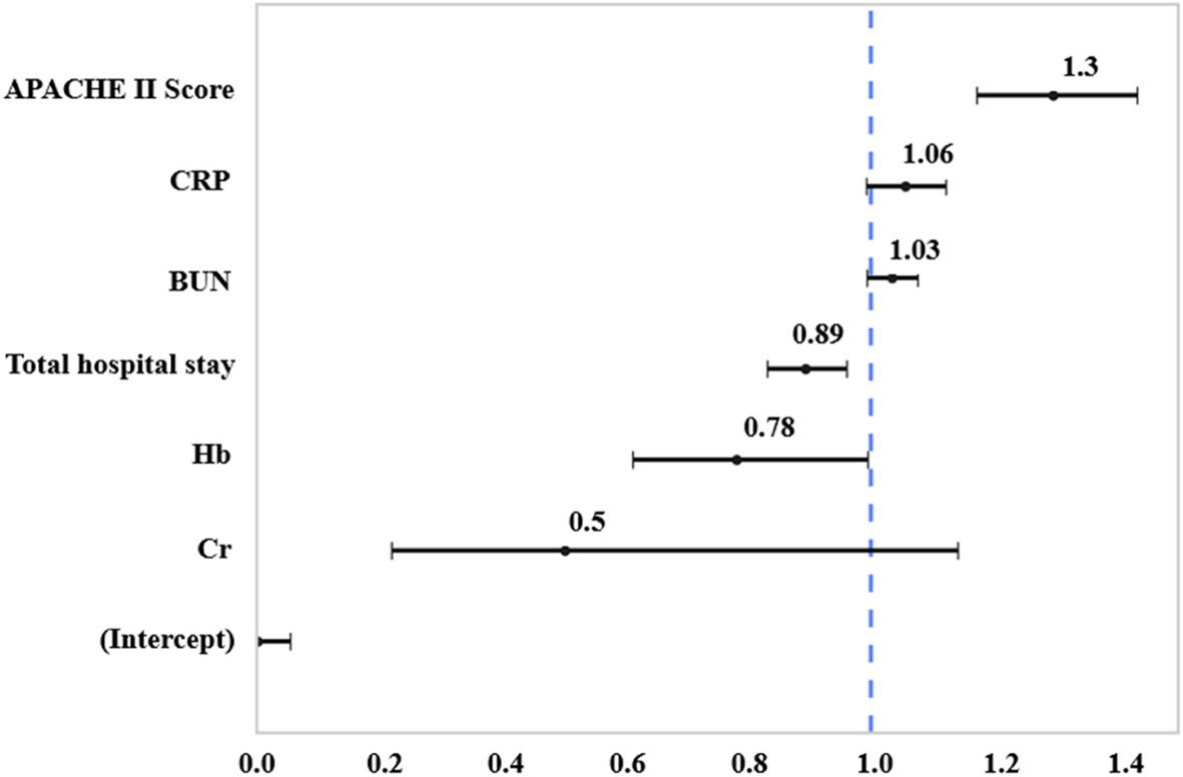
Multivariable logistic regression analysis of factors influencing 30-day mortality after surgery for gastrointestinal perforation

The multivariable logistic regression analysis revealed that a higher APACHE score (OR, 1.3; *P* < 0.001; 95% CI: 1.17–1.43) and longer total hospital stays (OR, 0.89; *P* = 0.005; 95% CI: 0.83–0.96) were independent and significant indicators for postoperative 30-day mortality (Fig. 3).

**Fig. 4:**
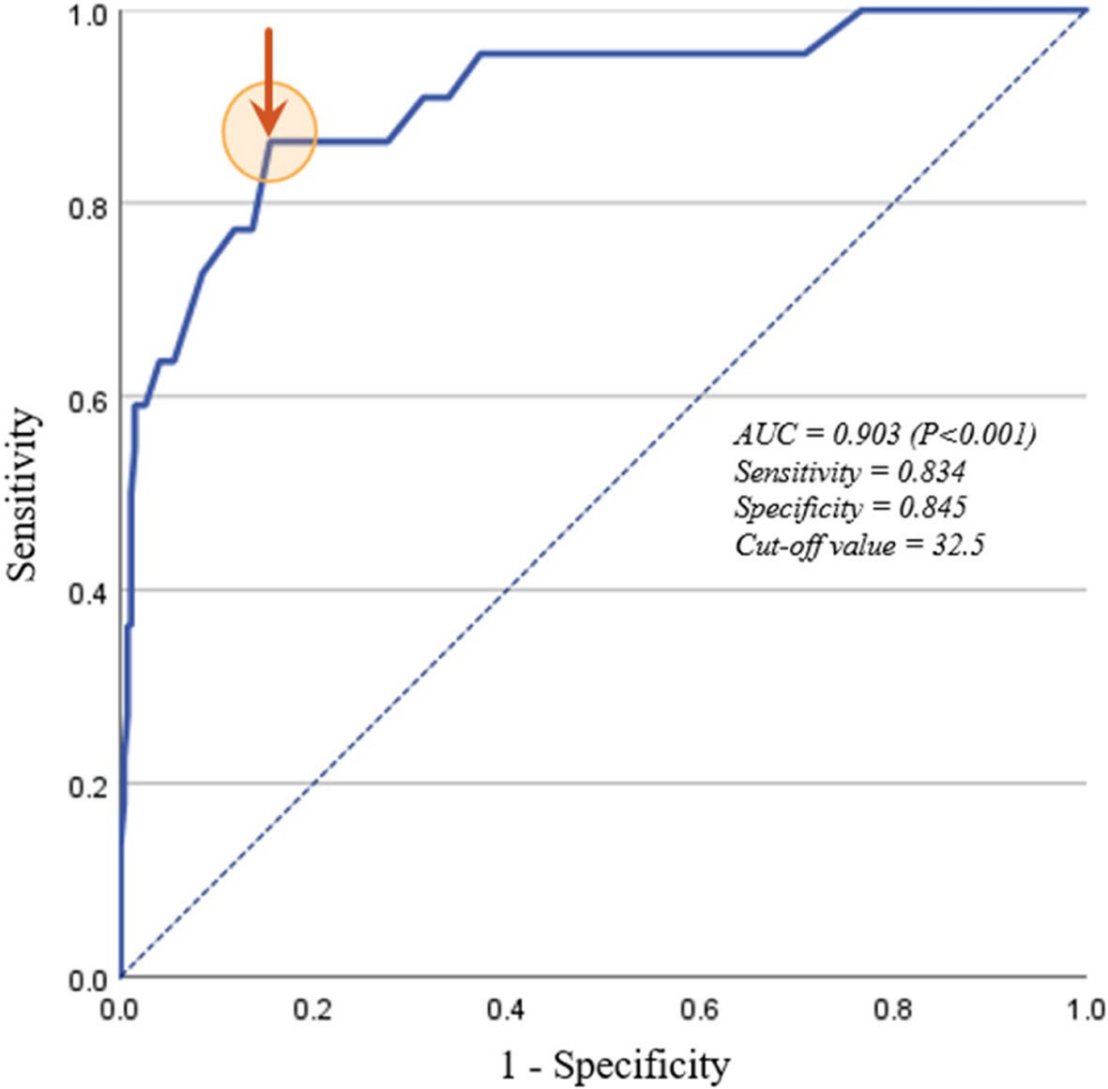
The area under the curve (AUC) and the optimal cut off value of APACHE II score. *APACHE, acute physiology and chronic helath evaluation

In receiver operating characteristic analysis, the area under the curve (AUC) of APACHE II score were 0.903 (95% CI: 0.835 to 0.980). The optimal cut off value of APACHE II score was 32.5 (Fig. 4).

**Fig. 5:**
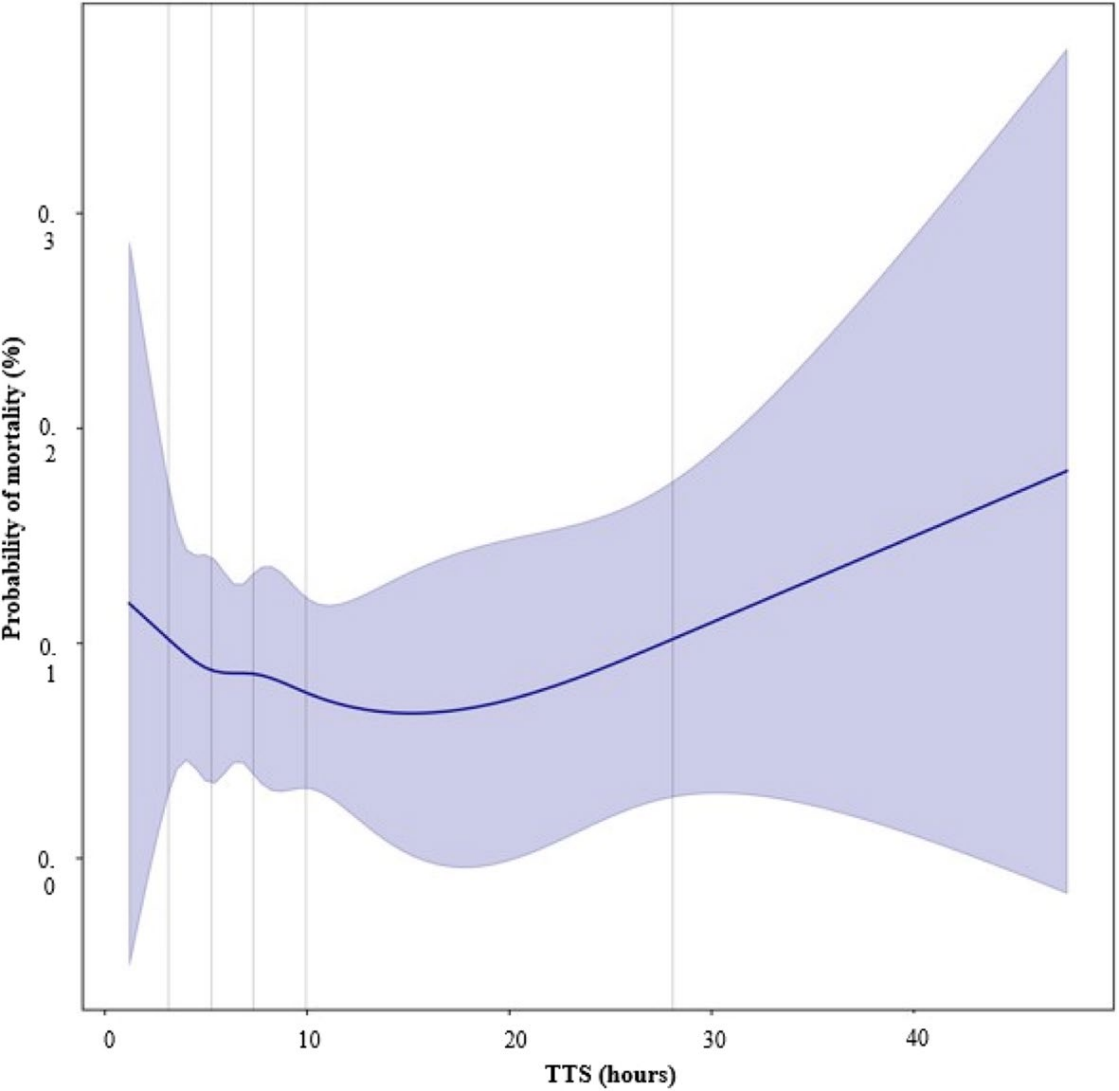
Restricted cubic spline curve (RCS) for probability of mortality rates over time from admission to surgery (TTS). Solid lines represent the estimated adjusted probability of mortality, and shaded bends represent 95% confidence intervals

Using the restricted cubic spline curve, the predictive value of mortality temporarily increased at the beginning when the TTS was only approximately 1–2 h. Subsequently, the mortality rate continued to decrease until a waiting time of 16 h. The curve began to ascend at 16 h as the diverging point and continued to increase thereafter (Fig. 5).

Since the mortality rate increased at 16 h after TTS in the RCS curve model, a subgroup analysis using propensity score matching was performed by dividing the patients into two groups based on a waiting time of 16 h. Table 2 presents the clinical characteristics and outcomes between the two groups. Using the propensity-matched cohort, no significant differences were observed between the two groups regarding surgical outcomes: 30-day mortality (11.4% versus 5.7%; *P* = 0.669); ICU stay (4.3 ± 7.5 versus 4.3 ± 5.2, *P* = 0.985); and total hospital stay (17.4 ± 17.0 versus 24.7 ± 23.4, *P* = 0.140); and transfer from other hospitals (45.7% versus 34.3%; *P* = 0.464). However, the patients who waited over 16 h group before surgery had a significantly higher rate of readmission to the ICU (3 (8.6%) versus 11 (31.4%); *P* = 0.036) compared with the under 16 h group.

**Table 2:** Comparisons of the covariates using subgroup analysis after propensity score matching

Variables	TTS over 16 h	TTS under 16 h	*P*-value
	(*n* = 35)	(*n* = 35)	
Sex (Male)	21 (60.0%)	13 (37.1%)	0.094
BMI	23.4 ± 3.1	21.7 ± 3.5	0.036
Operation time (h)	2.3 ± 1.28	2.61 ± 1.11	0.282
30-day mortality	4 (11.4%)	2 (5.7%)	0.669
ICU stay (days)	4.3 ± 7.5	4.3 ± 5.2	0.985
Total hospital stay (days)	17.4 ± 17.0	24.7 ± 23.4	0.140
ICU readmission	3 (8.6%)	11 (31.4%)	0.036
Anatomical location of the perforation site			
UGI	14 (40.0%)	13 (37.1%)	1.000
Small bowel	5 (14.3%)	9 (25.7%)	0.370
LGI	16 (45.7%)	15 (42.9%)	1.000
Lactate (mmol/L)	2.6 ± 2.9	3.5 ± 3.9	0.340
Hb (g/dL)	12.7 ± 2.1	11.3 ± 2.7	0.022
CRP (mg/dL)	8.6 ± 11.3	8.0 ± 8.7	0.792
Procalcitonin (ng/mL)	19.0 ± 34.6	3.5 ± 3.9	0.375
Transfer from other hospitals	16 (45.7%)	12 (34.3%)	0.464

Data are presented as number (percentage) or mean (± standard deviation)

Abbreviations: *BMI* body mass index, *ICU* intensive care unit, *UGI* upper gastrointestinal, *LGI* lower gastrointestinal, *Hb* hemoglobin, *CRP* C-reactive protein, *TTS* time from admission to surgery

## Discussion

Using the RCS curve, two peaks could directly be observed, indicating a pattern in which the curve decreased and then increased again in the interval between them. Of note, the first peak was observed in the group of patients with the shortest TTS, and this could be explained by that patients with high severity and possible septic shock were more quickly scheduled for surgery but expired. Since the predicted mortality rate increased after 16 h of waiting, it could be suggested that emergency surgery in patients with GI perforation should not exceed 16 h. Using a subgroup analysis with PSM, TTS > 16 h was associated with a higher chance of readmission to the ICU within 48 h after initial ICU discharge [15]. ICU readmission was associated with poor outcomes such as increased hospital stay and mortality rate [16–21]. However, this study did not show any other significant differences in clinical outcomes, such as 30-day mortality and length of hospital stay. This finding may have been influenced by early death in patients with high disease severity, as described above. In addition, due to the high heterogeneity of critically ill patients with completely different pathophysiology, locations of GI perforations, shock status, operations, and surgeons, it was difficult to match the patients and controls. Furthermore, the number of matched pairs was too small (n = 16), making it difficult to interpret the significance.

This retrospective cohort study showed that the APACHE II score was the only significant and independent predictor of postoperative 30-day mortality. The APACHE II is a classification system used for determining disease severity on a scale of 0–71. It is one of the most widely used scoring systems in ICUs. Higher scores correspond to higher severity and mortality [22–25]. However, there are still few studies that validated the performance of the APACHE II score in the surgical ICU population in Korea [23]. In this study, the APACHE II score was measured after surgical source control was completed. Moreover, the patients with higher scores had significantly higher mortality rates. This indicated that patient prognosis could be predicted depending on how well preoperative sepsis was resuscitated and how well surgical source control was performed. Although the total hospital stay was also independently associated with mortality, it was excluded because the length of hospitalization was inevitably shortened if patient expired. Additionally, because this study aimed to assess predictable variables and the hospital stay could be measured at the end of treatment, it could not be used as a predictor of mortality. The operation time was relatively longer in the non-survivors group, which could be explained by two hypotheses. First, the non-survivors had high APACHE II scores, indicating that they were more likely to have poor intra-abdominal conditions. This may have extended the operation time. Second, hypothermia or coagulopathy could occur owing to the extended time of the open abdominal cavity, which may have increased mortality [26].

This study has several limitations. First, since this was a retrospective study, many confounders should have been controlled. Although PSM matching was performed to adjust for confounders, unmeasured confounders or selection biases may still exist. Second, this study was conducted at a single institution, and the results cannot be generalized to other hospital environments. Therefore, a multicenter cohort study with a large sample size is needed to confirm the validity of these conclusions.

In conclusion, we were able to find an inflection point of 16 h through the RCS curve technique. Although subgroup analysis could not reveal significant differences in mortality, this finding should be interpreted with caution, as the analysis did not incorporate adjustments for patients who underwent emergency surgery due to extremely severe and potentially fatal conditions. This oversight could skew the results, underestimating the impact of rapid surgical intervention on mortality outcomes.

## Data Availability

The datasets generated and/or analysed during the current study are not publicly available in accordance with the institution's patient information protection principles but are available from the corresponding author on reasonable request.
